# Flow Diverter-Assisted Coil Embolization of Blood Blister-Like Aneurysm Using Semi-deploying Technique

**DOI:** 10.3389/fneur.2020.625203

**Published:** 2021-01-13

**Authors:** Ping Zhang, Weiying Zhong, Tao Li, Xianjun Tan, Chao Chen, Mingxin Li, Zhonggang Li, Gang Li, Yunyan Wang

**Affiliations:** ^1^School of Basic Medical Sciences, Shandong University, Jinan, China; ^2^Department of Neurosurgery, Qilu Hospital, Cheeloo College of Medicine, Institute of Brain and Brain-Inspired Science, Shandong University, Jinan, China; ^3^Shandong Provincial Key Laboratory of Brain Function Remodeling, Qilu Hospital, Shandong University, Jinan, China; ^4^Department of Neurosurgery, The Fourth People's Hospital of Jinan, Jinan, China; ^5^Department of Neurosurgery, People's Hospital of Chiping City, Liaocheng, China; ^6^Department of Neurosurgery, People's Hospital of Linyi City, Linyi, China

**Keywords:** blood blister aneurysm, flow diversion, semi-deploying technique, coil embolization, efficacy and safety

## Abstract

Despite many therapeutic methods were utilized to treat blood blister-like aneurysms (BBAs), the optimal treatment approach has not yet been defined. This study presents the single center experience with BBAs treated with flow diverter-assisted coiling using semi-deploying technique, and discusses the efficacy and safety of the method. The patients with subarachnoid hemorrhages (SAH) due to BBAs and treated with Pipeline Flex Embolization Device (PED) between November 2015 and February 2019 in our hospital were retrospectively reviewed. Patient demographic data, timing of treatment, angiographic details, treatment techniques, clinical outcomes and follow-up results were recorded. Ten cases (6 women and 4 men) were enrolled. The mean age of patients was 50.7 years (range 40–61 years). The aneurysm size ranged from 2 × 1.7 mm to 4.5 × 3.8 mm. Seven patients were treated with PED assisted coil embolization using semi-deploying technique, and all of the aneurysms were totally obliterated at the follow up. One patient treated with PED assisted coil embolization suffered from parenchymal hemorrhage 3 days after the treatment, and another one patient also treated with PED and coil died of severe vasospasm 10 days after the treatment. There was no reruptured cases during the follow-up. Here we showed that PED assisted coil embolization using semi-deploying technique could be a technically safe and effective treatment for BBAs.

## Introduction

Blood blister-like aneurysms (BBAs) are a rare subset of intracranial aneurysms which arise from non-branching segments of the dorsal or anterior wall of the supraclinoidal internal carotid artery (ICA) (1). BBAs account for 0.3–1.7% among all intracranial aneurysms and up to 6.6% of ruptured cerebral aneurysms (2). Histologically, BBAs behave as pseudoaneurysms and are made up of a broad base with adventitia and/or thrombus lacking both of the intima and media (3). The high rates of mortality, recurrence and poor prognosis in patients with BBAs are a testament to the unmet medical need presented by these aneurysms.

A lot of techniques have been developed to treat BBAs, such as surgical clipping, clipping after wrapping, suturing, surgical trapping, stenting with or without the assist of coils. Unfortunately, there is no optimal treatment for BBAs based on the long-term follow-up (4). Due to their wide neck, small size, and a fragile fibrous wall, BBAs are a therapeutic challenge for both surgical and endovascular treatment (5).

Endoluminal reconstruction with flow diverter appears to be a promising option for the treatment of BBAs, since it offers better mid- to long-term outcome (6). With the aim to achieve rapid aneurysm occlusion, flow diverter-assisted coiling using “semi-deploying” technique was employed to embolize BBAs at our center. This technique was found to be safe and effective, and demonstrated encouraging results in the treatment of ruptured BBAs.

## Methods

### Patient Population

Data from all patients suffering from subarachnoid hemorrhage (SAH) caused by BBAs and subsequently treated with Pipeline Flex Embolization Device (PED) between November 2015 and February 2019 in our hospital were retrospectively reviewed under the approval of the local institutional review boards.

Digital subtraction angiography (DSA) was used to diagnose patients with BBAs. Based on the angiographic studies, BBAs were defined as small (<5 mm), shallow, broad-based aneurysms originating from unbranched sites on the dorsal wall of the supraclinoid ICA. We collected patient clinical data, demographic data including aneurysm characteristics, Hunt and Hess Grade, procedural details and outcomes. All enrolled patients were treated with PED.

### Embolization Using Semi-deploying Technique Procedure

All patients undergoing PED therapy were administrated 300 mg of aspirin and 300 mg of clopidogrel 2 h before endovascular treatment. In hybrid operating room, all procedures were performed under general endotracheal anesthesia. Heparin was routinely administered during the procedure.

For the cases treated with PED assisted with coils using semi-deploying technique, the right and left femoral arteries were canalized by a 5-French (F) and an 8-F femoral artery short sheaths, respectively. For the cases treated with PED only, procedures were performed through an 8-F femoral artery access. Through the left 8-F femoral artery short sheath, an 8-F Mach 1 guiding catheter (Boston scientific, California, USA) was placed at the proximal ICA. 5-F Navein guiding catheter (Medtronic, California, USA) was positioned at the petrosal segment of parent ICA *via* the 8-F Mach 1 guiding catheter. Digital subtraction angiography and 3D rotational angiograms were performed to reassure the size, formation and location of the aneurysm and the diameter of the parent artery, helping select and deploy PED. A Marksman microcatheter (Medtronic, California, USA) was placed to the M2 segment of the middle cerebral artery through the Navien guiding catheter. The coiling microcatheter (Echelon 10, Medtronic, Minnesota, USA) was brought up and positioned into the aneurysm over 5F guiding catheter placed at the petrosal segment of parent ICA through the right 5F femoral artery short sheath. *Via* the Echelon 10 microcatheter 1 or 2 loops of coil were advanced into the aneurysm first. Then the PED was gently semi-deployed under fluoroscopic guidance, which means the PED was partially expanded and covered part of the aneurysm neck. The Echelon 10 microcatheter was partly jailed in the aneurysm through the wedge-shaped space between the PED and parent artery. The coil was carefully embolized within the BBA while the PED was further deployed if the coil bulged into the ICA. Following compact and complete embolization with the aneurysm, the Echelon 10 microcatheter was slowly removed and the PED was thoroughly deployed. The expansion of the PED was confirmed by fluoroscopy and Dyna CT computed tomography angiography.

Dual antiplatelet therapy (aspirin 100 mg/day and clopidogrel 75 mg/day) was started after the procedure. Four days after the procedure, thromboela-stogram (TEG) was customarily performed, by which arachidonic acid inhibition rate (AA%) and ADP inhibition rate (ADP%) were recorded. An AA% <50% and an ADP% <30% were defined as resistance to aspirin and clopidogrel, respectively. If the AA% was <50%, aspirin dose was increased up to 200 mg/d. If the ADP% was <30%, clopidogrel was dosed up to 150 mg/d or switched to ticagrelor (90 mg twice daily). If the inhibition rate was more than 90%, the risk for hemorrhage is high. The dose was reduced for the safety of patients. Three days after modifications to antiplatelet regimen, if any, TEG was reassessed. Dual antiplatelet therapy was continued for ≥6 months after the procedure.

### Data Sources and Follow-Up

The information about patient age, gender, aneurysms, complications, procedure details and outcomes was collected from medical charts in our hospital. Follow-up was performed at 3, 6, and 12 months after the embolization. The modified Rankin Scale (mRS) scores were assessed on the follow-up. After the procedure and at the 6–12 months follow-up, aneurysm occlusion was elevated according to Raymond Roy Scale with DSA.

## Results

In the study period, 10 patients (4 males) that experienced SAH from BBAs and who were subsequently treated with PED were identified. Unenhanced cranial CT and DSA was used to diagnose patients with SAH and BBAs. The age of patients that suffered from SAH ranged from 40 to 61 years (mean 50.7 years, SD 7.1). The initial Hunt and Hess Grade was 1 in four patients, 2 in four patients, 3 in one patient and 4 in one patient (mean 1.9, SD 0.99). The mean of the number of days from occurrence of SAH to the placement of PED was 6.8 days (range from 3 to 16 days, SD 3.9). Aneurysm size ranged from 2 × 1.7 mm to 4.5 × 3.8 mm (Table 1).

**Table 1:** Baseline clinical and radiographic data of patients with BBAs.

**Patient**	**Location**	**Size (mm, Neck × Dome)**	**Hunt-Hess grade**	**Days to treatment**
1	Left ICA	3.3 × 2.4	1	16
2	Right ICA	4.5 × 3.8	1	7
3	Left ICA	2.0 × 1.7	2	8
4	Right ICA	4.2 × 3.7	2	4
5	Left ICA	2.3 × 2.1	1	9
6	Left ICA	4.3 × 3.2	2	4
7	Right ICA	1.8 × 2.9	3	3
8	Right ICA	2.2 × 1.9	4	5
9	Right ICA	4.3 × 2.1	1	4
10	Right ICA	4.0 × 3.8	2	8

*BBAs, blood blister-like aneurysms; ICA, internal carotid artery*.

In all cases the procedure was technically successful. Most patients (7/10) were treated with PED assisted coil embolization using semi-deploying technique, and 3 received PED only. All the patients were treated with a single PED. Overall, the immediate angiographic results showed that 6 of 10 BBAs were totally obliterated and all these patients were treated with PED-assisted coil embolization using semi-deploying technique. The average of Raymond Roy Scale was 1.7 ± 0.95. At discharge the mean mRS Score was 1.8 ± 1.7 (Table 2). Patient 3 suffered from parenchymal hemorrhage 3 days after the procedure. Fortunately, the patient had mRS Score of 1 at last follow-up. Patient 5 had left lower limb deep venous thrombosis 3 days after embolization. Patient 8 developed severe vasospasm on the first day after the treatment, and died 10 days later in spite of symptomatic treatment.

**Table 2:** Results of the treatment of BBAs.

**Patient**	**PED size (mm)**	**Adjunctive coils**	**Raymond Roy scale post-treatment**	**Periprocedural complications**	**mRS score at discharge**	**Raymond Roy scale (6–12 months)**	**mRS score (6–12 months)**
1	3.75 × 20	No	3	NO	0	Not available	0
2	4.0 × 18	No	3	NO	1	1	0
3	3.25 × 20	Yes	1	Parenchymal hemorrhage	3	1	1
4	4.0 × 20	Yes	1	NO	1	1	1
5	4.5 × 20	Yes	1	Lower limb deep venous thrombosis	2	1	0
6	3.75 × 18	Yes	1	NO	1	1	0
7	3.5 × 20	Yes	2	NO	2	1	0
8	3.5 × 18	Yes	1	Death	6	Death	6
9	4.0 × 20	No	3	NO	1	2	0
10	4.0 × 20	Yes	1	NO	1	1	0

*BBAs, blood blister-like aneurysms; mRS, modified Rankin Scale; PED, Pipeline Flex Embolization Device*.

Only Patient 1 who was treat with PED alone had not undergone DSA for follow-up imaging during the follow-up 6–12 months after the procedure. Among the patients with follow-up DSA, the BBAs of all patients except Patient 9 were totally obliterated. The mean Raymond Roy Scale was 1.1 ± 0.35. No permanent deficit occurred. Patient 3 and 4 complained of paroxysmal and mild headache, respectively. The mean mRS Score was 0.8 ± 1.9 including the dead case (Table 2).

### Typical Cases

**Figure 1:**
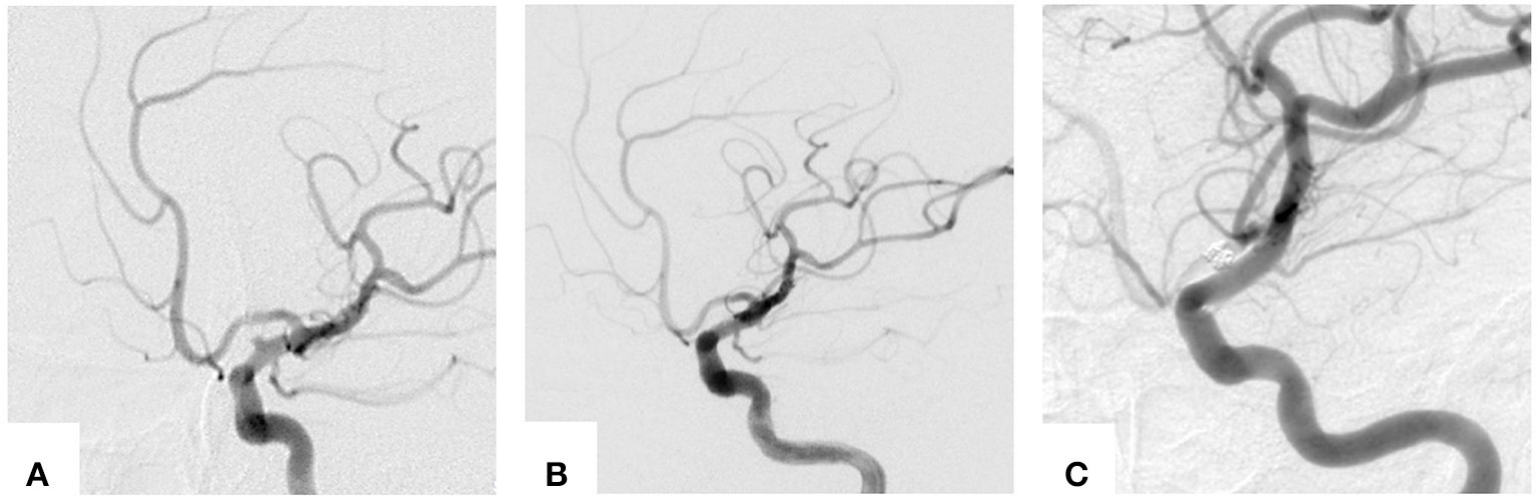
Images of the typical cases. **(A)** Cerebral angiograms obtained immediately pre-, **(B)** post-treatment and **(C)** 10-month follow-up DSA.

Patient 3: The patient presented with acute and severe headache accompanied by nausea (Hunt and Hess Grade 2). A CT scan at the local hospital demonstrated SAH. Five days later, the patient was transferred to our hospital where DSA was performed, exhibiting an aneurysm in the superior segment of the clinoid process of the left ICA (Figure 1A). The size of the aneurysm was 2.0 × 1.7 mm (neck × dome). Eight days post-SAH, the patient underwent PED-assisted coil embolization using semi-deploying technique in our hospital. One loop of coil (1.5 × 40 mm) was placed into the BBA after the Marksman microcatheter was positioned at the M2 segment of the left middle cerebral artery. Then the PED (3.25 × 20 mm) was gently semi-deployed, and two more coils (1.5 × 20 mm) were advanced. Subsequently, the PED was fully deployed. The aneurysm was completely occluded post-treatment (Figure 1B). A DSA examination performed 6 months after the procedure showed patent ICA without stenosis and no signs of BBA recurrence (Figure 1C).

**Figure 2:**
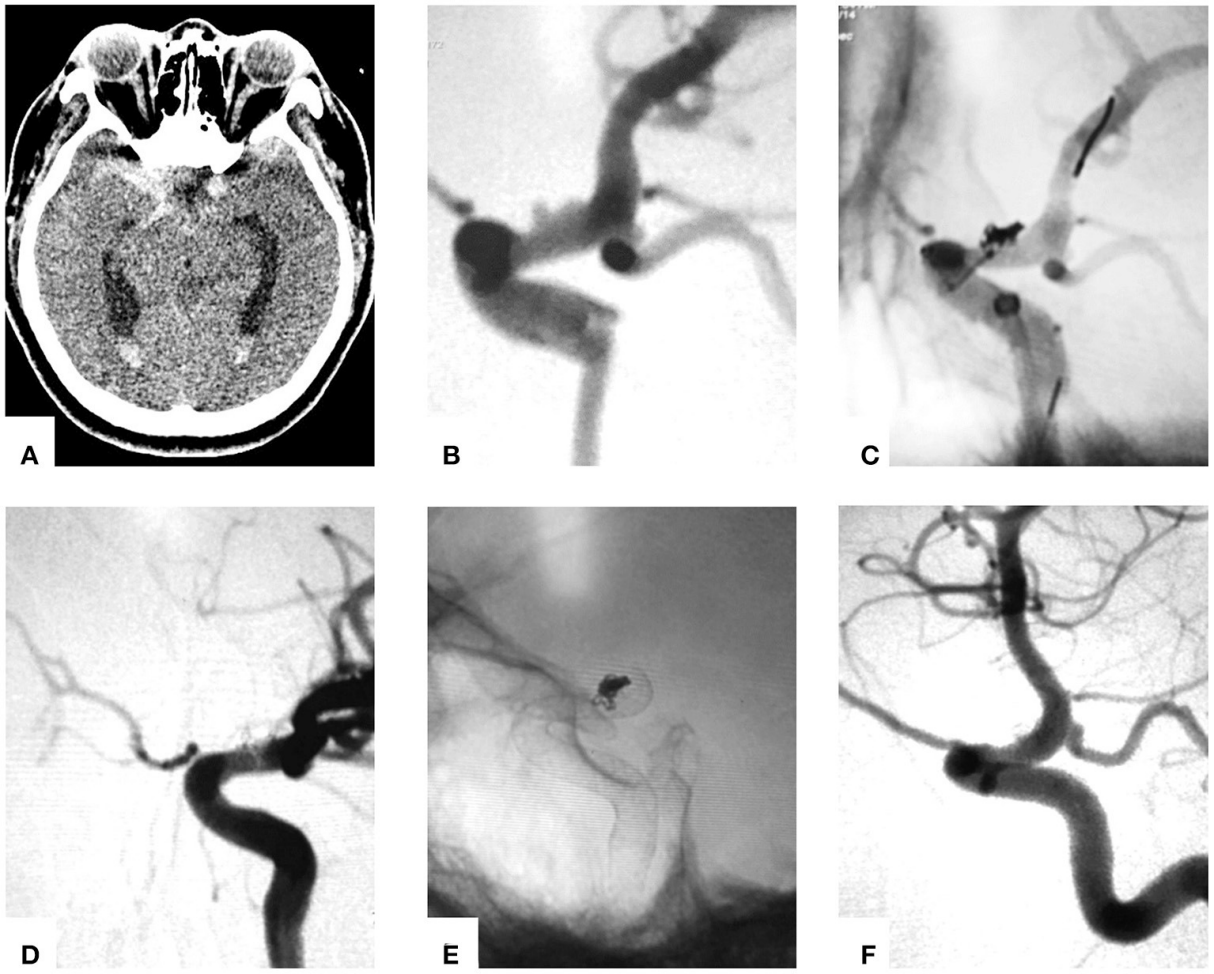
Images of the typical cases. **(A)** CT scan showed symmetric suprasellar cistern SAH and hemorrhage in lateral ventricles. **(B)** Cerebral angiograms obtained immediately pre-embolizaton. **(C)** PED semi-deployed coiling. **(D)** and **(E)** Post-treatment DSA. **(F)** DSA follow-up 6 months after treatment.

Patient 7: This patient presented with sudden onset of severe headache and vomiting (Hunt and Hess Grade 3), and a head CT scan showed SAH in suprasellar cistern and hemorrhage in lateral ventricles (Figure 2A). Digital subtraction angiography revealed a BBA (1.8 × 2.9 mm) of the supraclinoid right ICA (Figure 2B) 3 days after the occurrence of SAH. Then one-stage PED-assisted coil embolization was performed. First, the PED (3.50 × 20 mm) was gently semi-deployed, and three coils (1.5 × 40 mm, 1.0 × 40 mm, 1.5 × 40 mm) were delivered into the BBA. After complete embolization was achieved, the PED was fully deployed (Figure 2C). The aneurysm was completely occluded post-treatment (Figures 2D,E). Seven months follow-up DSA showed complete obliteration (Raymond Roy Scale 1) (Figure 2F).

## Discussion

Despite multiple therapeutic approaches (such as endovascular, microsurgical and hybrid therapy) are available to treat BBAs, satisfactory treatment of BBAs remains a challenge. Due to the rapid evolution of endovascular therapy within the last decade, embolization of BBAs may gain increasing acceptance in the future. Shah et al. systematically reviewed 36 papers involving 256 patients with BBAs treated with endovascular or surgical methods and concluded that endovascular therapy could offer lower morbidity and mortality (7).

Several endovascular approaches have been developed for treating BBAs, including direct coil embolization, multiple overlapping stents and flow diversion stents. Due to the thin and fragile characters of BBAs, most studies employing direct coil embolization of BBAs were performed with the assistance of a stent or balloon, and coiling alone was rarely reported as a sole treatment (8). Since direct coil embolization does not need antiplatelet medication it may be an appropriate choice for treatment of BBAs after the acute phase (9). The occlusion rates of ruptured BBAs treated with stent-assisted coiling were not encouraging: 33% initially post-procedure and about 70% at mid- to long-term follow-up (6). Fang et al. retrospectively reviewed 213 BBAs treated with stent-assisted coiling and reported that 64.6% of BBAs were completely obliterated and 22.9% recurred (10). Therefore, traditional coil embolization with or without stent is not the best alternative for treating BBAs.

Hao et al. declared endovascular patch embolization as an improvement on stent-assisted coil embolization and concluded that it could be an effective treatment for BBAs to facilitate flow guidance and embolic material stabilization (8). However, the study was limited by its small sample size (11). Multiple overlapping stents may be another workable option for treating ruptured BBAs. Theoretically, overlapping stents can improve flow diversion and reconstruct the fragile neck of the BBAs, reducing the risk of rerupture and recurrence (11). In the retrospective study, Fang et al. reported that placement of ≥ 2 stents resulted in higher complete obliteration rates and lower recurrence rates (10). Song et al. used multiple overlapping stents (≥ 3) with coiling in 10 patients with ruptured BBAs, presenting that 4 of the patients required complementary treatment (12). Gaughen et al. reported that 50% (3/6) of the patients treated with overlapping stents required retreatment for recurrence or residual (13). Some clinicians question whether overlapping stents are optimal for treating BBAs. Besides the unstable rates of aneurysm obliteration, dual antiplatelet administration which is necessary for overlapping stents treatment may increase the risk of post-operative rebleeding.

Flow diversion devices have the potential to be a standard therapy for BBAs, as confirmed by multiple studies (6, 14, 15). Through their dual-center experience, Linfante et al. concluded repairing BBAs with PED may be a safe and durable option as 9 out of 10 patients with ruptured BBAs were adequately treated with a single PED (15). Zhu et al. reviewed 165 patients with BBAs, claiming that PED could be safe and effective for BBAs, and a single PED offered higher rate of good outcomes compared with overlapped PED (16). But Marcus D. Mazur thought a single flow diverter might be incomplete for ruptured BBA because of their rebleeding case after the treatment with a single flow diverter (17).

In this study, we demonstrate flow diverter-assisted coil embolization of BBAs using semi-deploying technique. First, flow diversion with PED redirects blood flow away from the BBA, thereby reducing the risk of rebleeding and recurrence. Secondly, coils within the sac of BBAs could offer mechanical support during and after deployment, reducing the possibility of stent migration or foreshortening. Thirdly, semi-deployment of PED increases the flexibility and sensibility of the coiling catheter allowing improved control during the coiling procedure, thereby achieving a compact embolization in the BBAs.

This study evaluated 10 cases of BBAs, 7 of which were embolized with PED-assisted coil embolization using semi-deploying technique, and the other 3 were embolized with PED alone. Technical success was achieved in all cases with no intraoperative accident. One patient died of severe vasospasm, which may have been due to large amount of blood loss in the subarachnoid cavity. Because of high incidence of vasospasm of ICA and middle cerebral artery, intra-arterial infusion of nimodipine was used routinely, and it is recommended that balloon angioplasty should also be considered. Also due to vasospasm of ICA, the diameter of ICA may be underestimated on DSA imaging. So the diameter of flow diverter should be larger than the measured diameter of ICA. Another patient suffered from parenchymal hemorrhage which may have been caused by sensitivity to dual antiplatelet therapy. During the follow-up, except the dead case, 6 of 7 cases treated with PED assisted coil were obliterated. It has been proven that the process of PEDs healing aneurysms occurs progressively (18). Thereby, it is possible that the remnant would be occluded during the long term follow-up.

## Conclusion

The treatment of patients with ruptured BBAs by flow diverter-assisted coil embolization using semi-deploying technique resulted in good clinical outcomes. This therapy is emerging as a safe and effective alternative for the treatment of BBAs.

## Data Availability Statement

The original contributions generated in the study are included in the article/supplementary materials, further inquiries can be directed to the corresponding author.

## Ethics Statement

The studies involving human participants were reviewed and approved by the Ethics Committee of Qilu Hospital of Shandong University. The patients/participants provided their written informed consent to participate in this study. Written informed consent was obtained from the individual(s) for the publication of any potentially identifiable images or data included in this article.

## Author Contributions

PZ and YW designed the study and wrote the manuscript. WZ, TL, XT, CC, ML, and ZL collected the data and provided the interpretation of the data. GL reviewed the whole manuscript. All authors agree to be accountable for all aspects of the work, including its accuracy and integrity.

## Conflict of Interest

The authors declare that the research was conducted in the absence of any commercial or financial relationships that could be construed as a potential conflict of interest.

## Abbreviations

BBAs: blood blister-like aneurysms
DSA: Digital subtraction angiography
F: French
ICA: internal carotid artery
mRS: modified Rankin Scale
PED: Pipeline Flex Embolization Device
SAH: subarachnoid hemorrhages.

## References

[1] Abe M Tabuchi K Yokoyama H Uchino A. Blood blisterlike aneurysms of the internal carotid artery. J Neurosurg. (1998) 89:419–24. 10.3171/jns.1998.89.3.0419 9724116

[2] Peitz GW Sy CA Grandhi R. Endovascular treatment of blister aneurysms. Neurosurg Focus. (2017) 42:E12. 10.3171/2017.3.FOCUS1751 28565977

[3] Ishikawa T Nakamura N Houkin K Nomura M. Pathological consideration of a “blister-like” aneurysm at the superior wall of the internal carotid artery: case report. Neurosurgery. (1997) 40:403–5; discussion 405–6. 10.1097/00006123-199702000-00038 9007879

[4] Shah SS Gersey ZC Nuh M Ghonim HT Elhammady MS Peterson EC. Microsurgical versus endovascular interventions for blood-blister aneurysms of the internal carotid artery: systematic review of literature and meta-analysis on safety and efficacy. J Neurosurg. (2017) 127:1361–73. 10.3171/2016.9.JNS161526 28298019

[5] Ji T Guo Y Huang X Xu B Xu K Yu J. Current status of the treatment of blood blister-like aneurysms of the supraclinoid internal carotid artery: a review. Int J Med Sci. (2017) 14:390–402. 10.7150/ijms.17979 28553172 PMC5436482

[6] Rouchaud A Brinjikji W Cloft HJ Kallmes DF. Endovascular treatment of ruptured blister-like aneurysms: a systematic review and meta-analysis with focus on deconstructive versus reconstructive and flow-diverter treatments. Am J Neuroradiol. (2015) 36:2331–9. 10.3174/ajnr.A4438 26381557 PMC7964271

[7] Fang YB Li Q Yang PF Huang QH Zhao WY Xu Y. Treatment of blood blister-like aneurysms of the internal carotid artery with stent-assisted coil embolization. Clin Neurol Neurosurg. (2013) 115:920–5. 10.1016/j.clineuro.2012.09.022 23041379

[8] Hao X Li G Ren J Li J He C Zhang HQ. Endovascular patch embolization for blood blister-like aneurysms in dorsal segment of internal carotid artery. World Neurosurg. (2018) 113:26–32. 10.1016/j.wneu.2018.01.014 29325955

[9] Haji FA Boulton MR de Ribaupierre S. Blister-like supraclinoid internal carotid artery pseudoaneurysm in a 15-year-old male: case report and review of the literature. Pediatr Neurosurg. (2011) 47:449–54. 10.1159/000339355 22777210

[10] Fang Y Zhu D Peng Y Zhong M Xu J Li Q. Treatment of blood blister-like aneurysms with stent-assisted coiling: a retrospective multicenter study. World Neurosurg. (2019) 126:e486–91. 10.1016/j.wneu.2019.02.076 30825633

[11] Lee BH Kim BM Park MS Park SI Chung EC Suh SH. Reconstructive endovascular treatment of ruptured blood blister-like aneurysms of the internal carotid artery. J Neurosurg. (2009) 110:431–6. 10.3171/2008.7.JNS08257 19046039

[12] Song J Oh S Kim MJ Chung J Lim YC Kim BS. Endovascular treatment of ruptured blood blister-like aneurysms with multiple (>/=3) overlapping Enterprise stents and coiling. Acta Neurochir (Wien). (2016) 158:803–9. 10.1007/s00701-016-2721-8 26858206

[13] Gaughen JR Jr. Hasan D Dumont AS Jensen ME McKenzie J Evans AJ. The efficacy of endovascular stenting in the treatment of supraclinoid internal carotid artery blister aneurysms using a stent-in-stent technique. Am J Neuroradiol. (2010) 31:1132–8. 10.3174/ajnr.A2016 20150303 PMC7963918

[14] Aydin K Arat A Sencer S Hakyemez B Barburoglu M Sencer A. Treatment of ruptured blood blister-like aneurysms with flow diverter SILK stents. J Neurointerv Surg. (2015) 7:202–9. 10.1136/neurintsurg-2013-011090 24491271

[15] Linfante I Mayich M Sonig A Fujimoto J Siddiqui A Dabus G. Flow diversion with Pipeline Embolic Device as treatment of subarachnoid hemorrhage secondary to blister aneurysms: dual-center experience and review of the literature. J Neurointerv Surg. (2017) 9:29–33. 10.1136/neurintsurg-2016-012287 27075485

[16] Zhu D Yan Y Zhao P Duan G Zhao R Liu J. Safety and efficacy of flow diverter treatment for blood blister-like aneurysm: a systematic review and meta-analysis. World Neurosurg. (2018) 118:e79–86. 10.1016/j.wneu.2018.06.123 29944999

[17] Mazur MD Taussky P MacDonald JD Park MS. Rerupture of a blister aneurysm after treatment with a single flow-diverting stent. Neurosurgery. (2016) 79:E634–8. 10.1227/NEU.0000000000001412 27759680

[18] Becske T Brinjikji W Potts MB Kallmes DF Shapiro M Moran CJ Long-term clinical and angiographic outcomes following pipeline embolization device treatment of complex internal carotid artery aneurysms: five-year results of the pipeline for uncoilable or failed aneurysms trial. Neurosurgery. (2017) 80:40–8. 10.1093/neuros/nyw014 28362885

